# Hypertension in Women Across the Life Cycle: Unique Aspects and Challenges

**DOI:** 10.1007/s11906-026-01362-x

**Published:** 2026-02-20

**Authors:** Rachel M. Bond, Vikramjit Purewal, Natalie Cameron, Kardie Tobb, Demilade Adedinsewo, Ijeoma Isiadinso, Kameelah Phillips

**Affiliations:** 1 Women’s Heart Health, Dignity Health, Internal Medicine, Creighton University School of Medicine, Chandler, AZ USA; 2 HonorHealth Four Peaks Medical Center, Phoenix, AZ USA; 3 Department of Medicine (General Internal Medicine) and Department of Preventive Medicine, Northwestern University Feinberg School of Medicine, Chicago, IL USA; 4 Medical Director Women’s Heart & CardioObstetrics Program, Cone Health, Greensboro, NC USA; 5 Department of Cardiovascular Medicine, Mayo Clinic, Jacksonville, FL USA; 6 Department of Cardiovascular Medicine, Emory School of Medicine, Atlanta, Ga USA; 7 Calla Women’s Health, New York, NY USA

**Keywords:** Hypertension in women, Sex-specific cardiovascular risk, Hypertensive disorders of pregnancy, Menopause and blood pressure, Psychosocial stress and hypertension, Hormonal influences on cardiovascular health

## Abstract

**Background:**

Hypertension is a major driver of cardiovascular morbidity and mortality in women, with risk trajectories that evolve across the female life course. From reproductive years through menopause, sex-specific biological, hormonal, and social factors contribute to unique patterns of blood pressure risk and cardiovascular vulnerability.

**Objective:**

To provide a life-course review of hypertension in adult women and highlight stage-specific risk factors, clinical considerations, and opportunities for prevention and management.

**Content:**

Women experience distinct exposures that influence hypertension risk, including hypertensive disorders of pregnancy, adverse pregnancy outcomes, psychosocial stressors, cardiometabolic changes, and the transition through menopause. This review integrates key recommendations from the 2025 American College of Cardiology/American Heart Association High Blood Pressure Guideline and emphasizes practical, stage-specific approaches to screening, risk stratification, and treatment tailored to women.

**Conclusions:**

A life-stage–specific, sex-informed approach to hypertension is essential to improve early identification, optimize treatment, and reduce long-term cardiovascular risk in women. Addressing persistent evidence gaps and prioritizing implementation of sex-specific care strategies will be critical to advancing equitable cardiovascular outcomes.

## Introduction

Hypertension (HTN) remains a major public health issue due to its critical role in the development of cardiovascular disease (CVD), the leading cause of death globally [1, 2]. In the United States (U.S.), the economic impact of HTN is considerable, with healthcare costs estimated at $219 billion and 817 million health care events in 2019 [2]. Nearly half of U.S. adults have high blood pressure (HBP), defined as > 130/80 mmHg [1], and about half of these cases are uncontrolled. Geographic disparities persist: in 2022, Mississippi had the highest prevalence of HTN in US adults (40.2%), while Colorado had the lowest (24.6%). Prevalence of HTN increases with age, affecting 28.5% of adults aged 20–44, 58.6% of those 45–64, and 76.5% among those ≥ 65 [3].

Disparities in HTN are evidence across sex, race, and socioeconomic status. Racial disparities are shaped by both biological susceptibility and structural inequities, while socioeconomic disparities arise predominately from differential access to healthcare, health literacy and environmental determinants. While global prevalence between men and women is similar (~ 31%) [4], important age-related and racial differences are evident in the U.S. Prior to menopause, women generally have lower incidence of HTN compared to men; however, this advantage disappears with age. Data from the National Health and Nutrition Examination Survey (NHANES) 2011–2014 demonstrated that among US adults ≥ 65 years, 81.2% of women were hypertensive, compared to 73.4% of men [4]. Racial disparities are also striking: one study reported HTN prevalence in 22.3% of Black women of reproductive age, compared with 14.4% of White women and 9.0% of Hispanic women [5], underscoring the intersectional burden of risk.

The pathophysiology of HTN in women is multifactorial, shaped by dynamic mechanisms throughout the life course. Endogenous estrogen is thought to confer endothelial-protective effects, possibly accounting for the lower HTN prevalence observed in younger women. However, this relationship remains incompletely understood. Clinical studies indicate that exogenous estrogen may increase HTN risk in postmenopausal women [6], pointing to additional biological pathways. Furthermore, estrogen status alone does not account for persistent racial disparities in HTN prevalence among women of similar age groups.

Given the rising burden of CVD in women, a comprehensive understanding of the contributors to HTN across the adult lifespan is increasingly essential. Although HBP can first emerge during adolescence, this review focuses on adult women, recognizing its far-reaching impact from the reproductive years through menopause and later life. Blood pressure trajectories are shaped by life stage–specific factors such as hormonal transitions, psychosocial stress, comorbid conditions, and socioeconomic challenges, all of which differ from early adulthood to postmenopause. Integrating insights on race, gender, and social drivers of health (SDOH) is essential for developing meaningful and equitable approaches to care. In this review, we not only examine these sex-specific and life-stage-specific contributors but also highlight key insights from the 2025 American College of Cardiology/American Heart Association (ACC/AHA) *Guideline for the Prevention, Detection, Evaluation, and Management of High Blood Pressure in Adults* guideline. Finally, we identify critical research gaps and emerging therapeutic strategies that hold promise for reducing the disproportionate impact of CVD in women and for advancing sex-specific HTN care.

## Hypertension in Reproductive-age Women

### Unique Considerations

#### Oral Contraceptive Pills and Blood Pressure

Oral contraceptive pills (OCPs) are used by approximately 150 million women globally [7]. Beyond contraception, they treat menstrual cycle disorders, polycystic ovarian syndrome (PCOS), acne and hirsutism [8, 9]. Two major types exist: progesterone only pills and combined hormonal contraception (CHC) with estrogen and progestin [8]. Most CHCs contain ethinyl estradiol (10–50 mcg), while formulations with natural estrogens are less common [10].

Epidemiologic data from the U.S., England, Germany and Korea report higher BP among OCP users, with systolic increases of 0.7–5.8 mmHg and diastolic increases of 0.4–3.6 mmHg [10–14]. Duration of use and baseline cardiovascular (CV) risk (e.g., age, obesity, family history) appear to amplify this effect [10, 15–17]. In women with HTN, CHCs significantly raise CVD risk–a meta-analysis found a ninefold increase in myocardial infarction (MI) odds [18]. Ethinyl estradiol may elevate BP through enhanced angiotensinogen production and activation of the renin–angiotensin–aldosterone system (RAAS) [10, 19–21]. Progestin-only pills are not associated with HBP [22]. Though individual progestins may influence BP via androgen and estrogen receptor effects, BP differences between CHCs with various progestins are not clinically significant [23]. One exception is drosperinone, which has anti-mineralocorticoid properties and may lower BP when used in CHC or alone [24–27].

Accordingly, the World Health Organization (WHO) Medical Eligibility Criteria (MEC) advises against CHC use in women with HTN or major CVD risk factors such as age, tobacco use, diabetes mellitus (DM), or hyperlipidemia (HLD) [28]. The American College of Obstetricians and Gynecologists (ACOG) and the Centers for Disease Control and Prevention (CDC) recommend BP screening before CHC initiation, and routine monitoring. Progestin-only pills are generally safe and do not require additional monitoring [29, 30].

#### Polycystic Ovarian Syndrome and Hypertension Risk

PCOS affects 5–10% of women worldwide [31, 32]. Its associated metabolic dysfunction increases risk for insulin resistance, obesity, HTN and metabolic syndrome [33]. Meta-analyses report HTN prevalence of ~ 15% among reproductive-age women and 49% among postmenopausal women with PCOS; the risk remains 1.3–1.6 times higher even after adjusting for obesity [34–38]. This risk may be further increased with coexisting DM or HLD [37].

Hyperandrogenism and insulin resistance promotes RAAS activation and sympathetic overactivity, leading to vasoconstriction and sodium retention [33, 39–42]. Vascular stiffness has also been observed in adolescents with PCOS [43].

Treatment involves lifestyle optimization, CHCs, metformin, and increasingly, glucagon-like peptide-1 (GLP-1) receptor agonists [31, 44–46]. Annual CVD risk factor monitoring is recommended [44]. Blood pressure management should follow general population guidelines, with spironolactone considered for hyperandrogenic symptoms [42].

#### Preconception Planning for Women with HTN

Chronic HTN increases the risk of adverse pregnancy outcomes (APOs), including preeclampsia, cesarean section, preterm birth, and stillbirth [47]. Optimizing BP and CV health prior to conception is therefore essential [48, 49], and current guidelines emphasize comprehensive preconception counseling (Table 1). In addition to the 2025 ACC/AHA HBP guideline [1], both the ACOG and the Society of Maternal Fetal Medicine (SMF) have updated their recommendations following the Chronic Hypertension and Pregnancy Study (CHAP) trial. This study showed that treating mild chronic HTN to < 140/90 mmHg during pregnancy significantly reduced preeclampsia and adverse perinatal outcomes without increasing the risk of small-for-gestational-age infants [50, 51]. These findings shifted prior guidance, which delayed treatment until BP exceeded ≥ 160/110 mmHg. Current recommendations now support initiating or adjusting antihypertensive therapy in women planning pregnancy if BP is ≥ 140/90 mmHg. Continued research is needed to refine optimal BP targets and treatment strategies during the preconception period to further improve maternal and fetal outcomes.

**Table 1:** Key components of the preconception visit for women with chronic HTN

Early Awareness	Obtain a detailed family history of CVD, HTN, and preeclampsia; review patient’s personal history of APOs
Screening for Secondary Causes of Hypertension	Consider in women with HTN onset < 30 years, resistant HTN, abrupt worsening of control, unprovoked/excessive hypokalemia, or signs/symptoms suggestive of secondary causes (e.g., Cushing’s syndrome, pheochromocytoma, aortic coarctation)
Laboratory Testing	Basic metabolic panel, complete blood count, thyroid-stimulating hormone, urinalysis, microalbumin-to-creatinine ratio, lipid profile
Electrocardiogram (ECG)	Assess for cardiac abnormalities
Lifestyle Counseling	Provide counseling on DASH or Mediterranean diet, regular physical activity, weight management, and cessation of tobacco and alcohol use; incorporate family-based lifestyle strategies when appropriate
Medication Review	Assess teratogenic risk of antihypertensive medications. Discontinue agents unsafe in pregnancy (e.g., ACE inhibitors, ARBs)
Aspirin Counseling	Discuss initiation of low-dose aspirin between 12–28 weeks of gestation
Sleep Apnea Screening	Consider screening based on clinical risk
Routine Preconception Counseling	Include immunizations, infectious disease screening, substance use, intimate partner violence, genetic screening, and folic acid supplementation

### Pregnancy and Hypertension

Hypertensive disorders of pregnancy (HDP), defined as BP ≥ 140/90 mmHg (severe ≥ 160/110 mmHg), include chronic HTN, gestational HTN, and preeclampsia (Table 2) [52, 53]. While HTN staging typically follows the ACC/AHA classification in nonpregnant populations, this system is not applied during pregnancy due to differing pathophysiology and treatment goals [1, 52]. Instead, HDP diagnosis and management require pregnancy-specific criteria, where BP should be assessed at every prenatal visit [54].

**Table 2:** Definitions of hypertensive disorders of pregnancy

Chronic Hypertension	Hypertension diagnosed before pregnancy, prior to 20 weeks of gestation or that persists more than 12-weeks postpartum
Gestational Hypertension	Hypertension diagnosed after 20 weeks of gestation; no evidence of end-organ damage
Preeclampsia	Hypertension diagnosed after 20 weeks of gestation and complicated by either proteinuria or one of the following: low platelets, kidney injury, liver dysfunction, pulmonary edema, headache that does not respond to usual medications or other neurological symptoms
Preeclampsia with Severe Features	Preeclampsia diagnosed after 20 weeks of gestation with BP ≥ 160/110 mmHg on two readings or low platelets, kidney injury, liver dysfunction, pulmonary edema or neurological symptoms
Hemolysis, Elevated Liver Enzymes, and Low Platelet Count (HELLP) Syndrome	Preeclampsia with hemolysis, AST and ALT elevations and platelet count < 100,000 cells/microliter
Eclampsia	New-onset seizures that occur in the absence of a secondary cause. Typically preceded by a HDP
Preeclampsia/Eclampsia Superimposed on Chronic HTN	Preeclampsia or eclampsia in women with a history of pre-existing or chronic HTN

Evaluation of new-onset HTN includes a complete blood count, comprehensive metabolic panel, and assessment for proteinuria [52]. Management thresholds differ from the nonpregnant population due to concerns that aggressive BP reduction may impair uteroplacental perfusion. As discussed earlier, chronic HTN is now treated at ≥ 140/90 mmHg to improve maternal and fetal outcomes [50, 51]. However, for gestational HTN or preeclampsia, U.S. guidelines still generally recommend initiating treatment at ≥ 160/110 mmHg, as updates incorporating newer evidence have not yet been adopted [52, 55]. Importantly, care should be individualized, and lower treatment thresholds may be appropriate for select patients depending on comorbidities, symptoms, and overall risk profile.

Recommended antihypertensive medications during pregnancy are listed in Table 3 [56, 57]. Aspirin is advised between 12 and 28 weeks gestation in women at risk of preeclampsia. This includes women with at least one high risk feature (history of preeclampsia, chronic HTN, pregestational DM, kidney disease, autoimmune disease, multifetal gestation) or ≥ 2 moderate risk features (age ≥ 35, BMI > 30, nulliparity, > 10-year pregnancy interval, in vitro fertilization, previous APOs, first degree relative with preeclampsia, Black race [proxy for racism], lower income) [56].

**Table 3:** Preferred blood pressure medications to use during pregnancy and lactation

First Line Agents During Pregnancy	labetalol nifedipine methyldopa
Second-Line Agents During Pregnancy	hydralazine chlorthalidone or hydrochlorothiazide Clonidine
Preferred Agents in Lactation	nifedipine labetalol hydrochlorothiazide^1^ hydralazine enalapril, captopril, benazepril^2^

1 – May decrease milk production

2 – Recommend close monitoring of infant’s weight and contraceptive counseling

HDP significantly increases both short- and long-term CVD risk. Within the first year postpartum, HDP contributes to ~ 7% of maternal deaths [57]. Persistent or newly diagnosed chronic HTN occurs in 25–50% within 1–2 years postpartum [58, 59]. HDP is also recognized as a risk-enhancing factor by the ACC/AHA, associated with a ~ twofold increased risk of coronary artery disease, heart failure and stroke [60–62]. Proposed mechanisms include inflammation, vascular remodeling, antiangiogenic factor dysregulation, and potentially the unmasking of preexisting CVD during pregnancy [61]. Ongoing research is needed to clarify the links between pre-pregnancy CV health, HDP and future CVD-risk.

Timely postpartum care is critical. Follow-up is recommended at 7–10 days postpartum– or within 72 h for those with severe HTN [52, 56]. Long-term care should include a primary care clinician (PCC) and/or cardiologist, with counseling on lifestyle modification and routine CVD risk screening [61, 63]. This includes guidance on healthy diet, physical activity, postpartum weight loss, and lactation, as well as screening for HLD, DM and persistent proteinuria [61, 63].

Recent studies have explored the relationship between HDP and breastfeeding practices. Breastfeeding is associated with reduced risks of maternal cardiometabolic disease, preeclampsia, and all-cause mortality in both mothers and infants, with longer duration potentially enhancing these benefits [64]. A 2025 cross-sectional investigation by Nardella et al. found HDP was linked to higher rates of never initiating breastfeeding and shorter duration [64]. These findings highlight another potential sequela of HDP: never initiating or early cessation of breastfeeding may contribute to increased risk of cardiometabolic disease, preeclampsia, and all-cause mortality. Importantly, breastfeeding support programs represent a modifiable intervention to help mitigate these risks.

Despite these benefits, postpartum care remains suboptimal, even when reinforced during the postpartum visits. As a call to action, the 2025 AHA/ACC High Blood Pressure Guideline [1] highlights the importance of early and ongoing BP management in the postpartum period, emphasizing lifestyle measure—including dietary modification, sodium reduction, physical activity, weight management, and reduced alcohol intake—to improve long-term outcomes. This renewed focus is timely, as fewer than 60% of women with HDP follow up with primary care in the first year postpartum, and only about half recall receiving counseling on diet or exercise [65, 66]. Closing these gaps will require intentional strategies such as timely appointment scheduling, patient navigation, home visits, and referral to dedicated postpartum cardiometabolic clinics staffed by multidisciplinary teams including obstetricians, PCCs, cardiologists and social workers [67].

## Hypertension in Midlife Women

### Pressure Points: Menopause, Hormones, and Hypertension

The transition through perimenopause and menopause represent a pivotal period of hormonal and CV change. Perimenopause, which spans 4–10 years before the final menstrual period (FMP), is marked by fluctuations in estradiol, progesterone, and testosterone. Menopause–clinically defined as 12 months of amenorrhea–signals the permanent cessation of ovarian hormone production [68]. In the U.S., menopause typically occurs between ages 42–58, with a median age of 52 years [69]. By 2025, more than 1.1 billion women globally will be postmenopausal [70].

Menopause contributes to unfavorable CV risk profiles, including increased visceral adiposity, endothelial dysfunction, systemic inflammation and shifts in lipid metabolism [71]. Blood pressure commonly rises within 5–20 years postmenopause, reflecting estradiol’s role in vascular tone and autonomic regulation [72]. As women now live nearly 40% of their lives postmenopause, this phase necessitates targeted HTN prevention strategies.

Although men tend to have higher rates of HTN earlier in adulthood, women experience a steeper rise in BP beginning in their 40 s, ultimately surpassing men by their seventh decade [73, 74]. By age 60, more than two-thirds of women have HTN, with postmenopausal women disproportionately affected by resistant HTN and lower rates of BP control [75, 76]. While this age-related increase in HTN is well established, midlife women encounter unique physiological transitions that complicate BP regulation and therapeutic response.

As previously noted in the context of OCP use and PCOS, estrogen is a key modulator of CV physiology. Menopause marks a critical turning point that accelerates CV risk, with the sharp decline in endogenous estrogen during perimenopause and postmenopause contributing to BP elevation. This risk is likely mediated by increased sympathetic tone, vascular stiffness, and RAAS activation. Supporting this, premature menopause–either naturally occurring or induced by bilateral oophorectomy or gonadotoxic therapies–is associated with an even greater risk for HTN and CVD, independent of traditional factors such as age, smoking or hormone replacement therapy (HRT) use [77–81].

In addition to hormone changes, midlife women experience increased salt sensitivity–a phenomenon that worsens with aging and the menopause transition. This heightened sensitivity is independent of aldosterone levels or a prior HTN diagnosis and leads to exaggerated BP responses to dietary sodium [82–84]. Compounding this, postmenopausal women demonstrate elevated oxidative stress, largely due to loss of estrogen’s vascular protective effects. Endogenous estrogens act as potent antioxidants, scavenging reactive oxygen species with an efficacy estimated to be 2.5 times greater than vitamins C or E, thereby conferring important vascular benefits during reproductive years [85].

### Hormone Replacement Therapy: Friend or Foe?

HRT is used to relieve moderate to severe vasomotor symptoms by replacing estrogen (and progestin if the uterus is intact). Hormones can be administered oral, transdermal, and/or vaginal [86].

While endogenous estrogen helps regulate BP, the effects of hormone therapy are more complex. Early observational studies, including the Nurses’ Health Study (NHS), suggested that HRT use was associated with a 40–50% lower risk of CVD and all-cause mortality [87–89]. However, subsequent randomized trials provide conflicting evidence. The Postmenopausal Estrogen/Progestin Interventions (PEPI) trial, conducted in healthy postmenopausal women (mean age of 56), found no significant BP effect from oral estrogen, either alone or combination with progestin–a finding supported by other studies [90]. Importantly, long-term HRT does not reliably restore or maintain premenopausal BP levels, making it unlikely to serve as a viable long-term BP management strategy.

The Heart and Estrogen/Progestin Replacement Study (HERS), the largest randomized trial of HRT in women with heart disease (mean age 66.7), showed a small rise in systolic BP (1–2 mmHg) without reducing major CV events [91]. The landmark Women’s Health Initiative also found a modest increase in BP among those using oral conjugated equine estrogens (CEE). A key insight from later analyses, often missed in public discussion, was the "timing hypothesis": the effects of HRT depend on age (particularly ≤ 60 years), time since menopause (within 10 years of menopause onset), and underlying health comorbidities [92].

Worldwide, estrogen options include CEE, estradiol (E2), and tibolone–a synthetic steroid that acts as an agonist primarily at estrogen receptors (ER); however, tibolone is not available in the US because of evidence linking its use in postmenopausal women to an increased risk of stroke and breast cancer. CEE, derived from pregnant mares’ urine, raises BP more than E2, especially when taken orally due to first-pass metabolism [93–95]. Transdermal E2, which bypasses hepatic metabolism, has fewer BP effects and has a more favorable CV profile [96].

As previously mentioned, the progestin used with estrogen also matters. Drospirenone, with antimineralocorticoid properties, may modestly lower BP. Other forms like micronized progesterone or dydrogesterone seem BP-neutral, though more research is needed [97, 98]. Meta-analyses show that transdermal E2 with progestin has minimal effect on BP, whereas oral CEE may modestly increase it [94, 99].

In summary, HRT’s effects on BP depend on hormone type, route of administration, when it’s started, and a woman’s baseline CV risk. While oral CEE may slightly increase BP, transdermal E2–with or without progestins–appears safer for women with or at risk for HTN. As more women enter midlife with HBP, careful and individualized use of HRT is vital, with CV risk assessment as a key part of care.

### Lifestyle & Behavioral Factors Affecting Blood Pressure in Menopause

#### Obesity, Physical Inactivity, Dietary Factors, and Salt Sensitivity

The menopause transition is associated with phenotypic changes, including increased weight, visceral fat, and central obesity. On average, women gain about one pound per year–often adding up to more than 10 pounds during the transition–due to falling estrogen levels, slower metabolism, and more insulin resistance [100–104]. Visceral fat, which rises from 5–8% to 15–20% of total body fat after menopause, is linked to a higher risk of metabolic syndrome, DM, and CVD [105]. Central obesity also raises BP through insulin resistance, increased sympathetic activity, and activation of the RAAS [85, 106, 107].

Physical activity frequently declines in midlife, influenced by factors such as fatigue, musculoskeletal discomfort, caregiving responsibilities, mood disturbances, worsening vascular function and stress regulation [108]. Regular aerobic and resistance training improve endothelial function and support nitric oxide (NO)–mediated vasodilation [109]. Physical activity remains a core, non-pharmacological strategy for HTN prevention and management in menopausal women [110, 111]. Studies show it can lower systolic BP by 6–7 mmHg and diastolic BP by 3–4 mmHg [112–116]. Even low-cost, accessible forms of aerobic activity, such as walking, have been shown to effectively lower BP across all age groups, including after menopause [117].

In addition, postmenopausal women exhibit increased salt sensitivity due to diminished estrogen-mediated vasodilation, leading to exaggerated BP responses to sodium intake [118, 119]. Even modest increases in sodium can elevate BP, and diets high in processed foods and low in fresh fruits and vegetables–characteristics of the Western dietary pattern–further exacerbate this effect. By contrast, the Dietary Approaches to Stop Hypertension (DASH) and Mediterranean diets, particularly with sodium intake below 1,500 mg/day, have been shown to consistently lower BP and improve CV outcomes [82, 120]. The 2025 AHA/ACC HBP Guideline reinforces these findings, emphasizing sodium restrictions to < 1,500 mg/day and recommending adequate dietary potassium intake (3,500–5,000 mg/day) as complementary strategies to improve BP control and reduce cardiovascular risk [1].

#### Lifestyle and Behavioral Considerations

Beyond biology, psychosocial stress plays a key role in HTN. Stress activates the hypothalamic–pituitary–adrenal (HPA) axis and sympathetic nervous system, raising cortisol levels and sympathetic tone. Menopause is a time of vulnerability due to workplace pressures, caregiving burdens, and social stressors, all of which disrupt healthy routines and contribute to long-term strain on the body–known as allostatic load [121–126].

Occupational stress– driven by ageism, gender bias, high demand, and low autonomy– raises allostatic load and increases HTN risk. It worsens sleep, promotes maladaptive behaviors, and perpetuates HPA axis hyperactivation, raising HTN risk. Many midlife women are also “sandwich generation” caregivers, juggling responsibilities for both children and aging parents. Caregiving for ≥ 15 h/ week is linked to double the rate of moderate-to-severe menopause symptoms and a 36–47% rise in HTN [127]. Caregivers often neglect their own health, with less physical activity, worse diets, and more tobacco use–worsening cardiometabolic outcomes.

These burdens are heavier for Black women, who face cultural pressure to perform across multiple roles while avoiding vulnerability–the “superwoman schema”. This, combined with the chronic stress of racism, discrimination, and caregiving, increases weathering–a biological aging effect caused by long-term exposure to stress. It leads to higher BP and worse CV outcomes [128–130]. Discrimination in healthcare and the workplace deepens these gaps.

Effective strategies exist. Cognitive behavior therapy, mindfulness, and stress-resilience programs help lower BP and reduce anxiety [131]. Social support–peer groups, therapy and caregiver resources—buffers chronic strain. Policies supporting workplace flexibility and caregiving protections may further ease the burden [132].

**Fig. 1:**
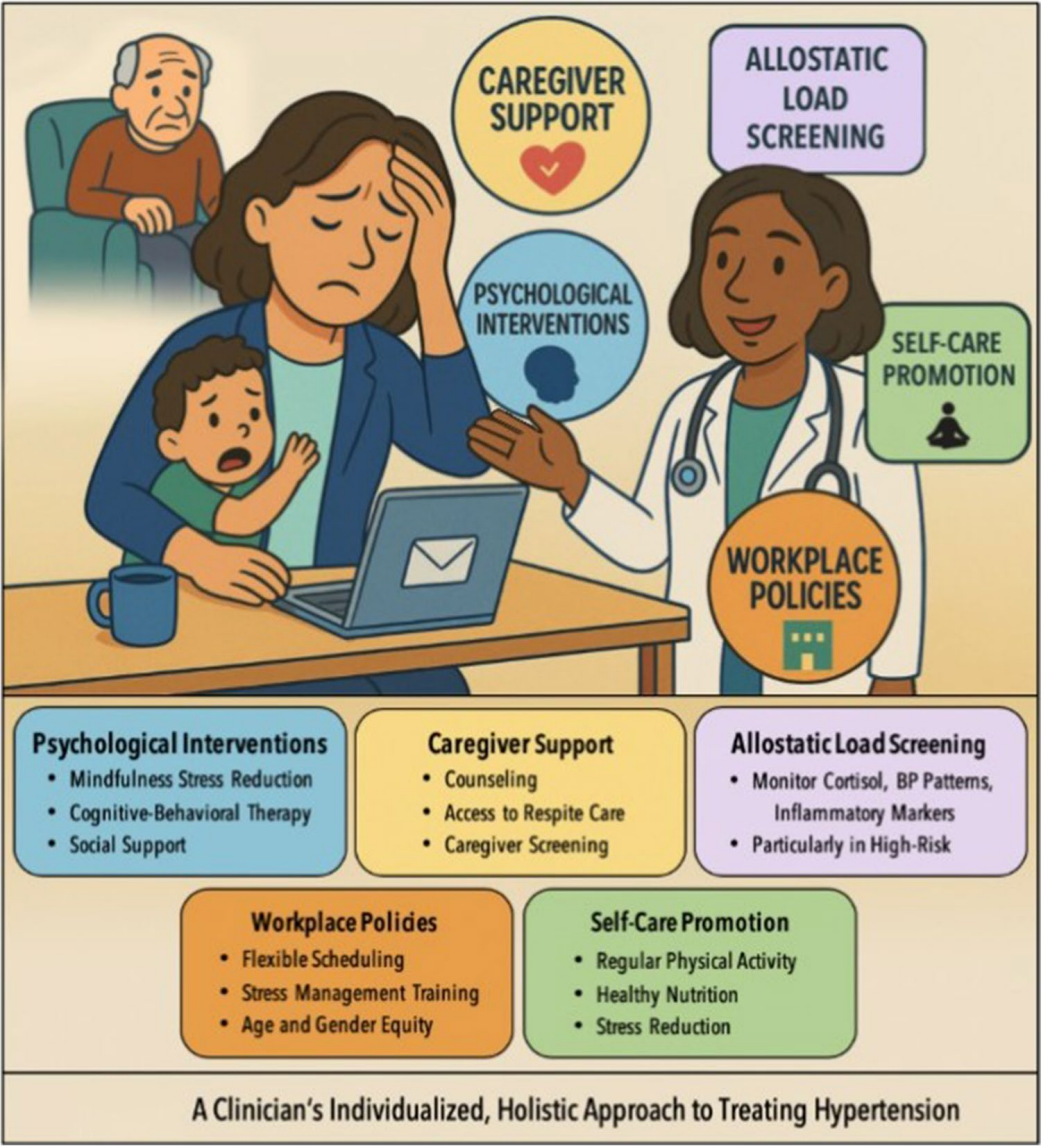
A Clinician’s individualized, holistic approach to treating hypertension

Clinicians should take an equity-informed, intersectional approach to treating HTN in midlife women–especially women of color (Fig. 1). This means considering how race, gender, and life experience shape health risks. Emphasis should be placed on physical activity, nutrition, sleep, stress support, and connection to the community [133, 134]. Tracking stress-related biomarkers like cortisol, high density lipoprotein, total cholesterol, Hemoglobin A1c, and C-reactive peptide may identify high-risk individuals for early intervention [135].

## Hypertension in Older Women

### Aging and Vascular Changes

As previously discussed, women experience a steeper rise in BP beginning in their 40 s, ultimately surpassing men by the seventh decade of life [4, 136]. In older age, this trend continues, with women more likely to have HTN and less likely to have it well-controlled. Data from NHANES 2017 to 2020 show that although HTN is more prevalent in men up to age 64, women become disproportionately affected thereafter [136]. Isolated systolic hypertension defined as systolic BP ≥ 130 mmHg with diastolic BP < 80 mmHg is particularly common in older women, especially after menopause, primarily due to decreased vascular compliance.

While hormonal shifts during the menopause transition contribute to early vascular aging, aging itself accelerates these processes. Older women exhibit increased arterial stiffness, impaired endothelial function, and reduced NO availability, all of which contribute to sustained BP elevation. These changes are often more pronounced in women than in men, likely due to sex-specific differences in vascular structure, hormonal history, and longevity. The result is not only higher HTN prevalence in older women, but also a distinct vascular phenotype that requires tailored diagnostic and treatment strategies [137].

### Pharmacologic Management Challenges

Given the rising burden of HTN with age and its associated CV morbidity and mortality, effective BP control in older adults is essential. The 2025 AHA/ACC High Blood Pressure Guideline recommends initiating pharmacotherapy for higher-risk adults with HBP ≥ 130/80 mmHg, without setting specific thresholds based on age or sex [1]. First-line therapies include dihydropyridine (DHP) calcium channel blockers (CCBs), thiazide-like diuretics, angiotensin-converting enzyme inhibitors (ACEi), and angiotensinogen receptor blockers (ARBs), but special considerations are warranted when treating older women [138].

The Systolic BP Intervention Trial (SPRINT) demonstrated that intensive BP lowering (target systolic BP or SBP < 120 mmHg) significantly reduced fatal and nonfatal CV events compared with standard treatment (systolic BP < 140 mmHg) in adults aged ≥ 75 years (5.2% vs. 6.8%, hazard ratio [HR] 0.75, 95% confidence interval [CI] 0.64–0.89; *p* < 0.0001) [139]. Notably, there was no increase in orthostatic hypotension or injurious falls. However, generalizability is limited, as only 36% of the participants were women, limiting the applicability to older female populations–an age most importantly found to have isolated systolic hypertension [140].

The Hypertension in the Very Elderly Trial (HYVET), which enrolled 3,845 patients aged > 80 years (61% women), showed that indapamide with or without perindopril significantly reduced stroke, heart failure, and all-cause mortality compared to placebo [141].

Similarly, the 2021 Strategy of Blood Pressure Intervention in the Elderly Hypertensive Patients (STEP) trial, conducted in China, included 8,511 participants aged 60–80 years (~ 53% women) and demonstrated that intensive BP lowering (SBP 110–130 mmHg) reduced cardiovascular events compared with standard treatment (SBP 130–150 mmHg) [142]. The greatest representation of women in HYVET and STEP provided more balanced sex-specific analyses, though the exclusion of very frail individuals still limits broader applicability.

Together, these findings suggest stricter BP targets may be beneficial in older adults, though recommended goals remain variable (SBP < 150 mmHg to < 130 mmHg), depending on comorbidities, functional status, and individual tolerance (Table 4).

**Table 4:** Blood pressure targets based on hypertension trials and recent guidelines

Population	Target BP	Comments
General Adult Population (2025 AHA/ACC Guideline)	< 130/ < 80 mmHg	An age- or sex-specific threshold is not defined; first-line therapies include DHP CCBs, thiazide-like diuretics, ACEi, ARBs
General Adult Population (2024 ESC Guideline)	SBP 120–129 mmHg DBP 70–79 mmHg	A sex-specific threshold is not defined; for older patients (≥ 85 years) and those who do not tolerate the primary treatment target, the BP goal is "as low as reasonably achievable" (ALARA). First-line therapy for most patients involves a combination of an ACEi/ARB and a CCB or an ACEi/ARB and a thiazide
Adults > 75 years (SPRINT trial)*	< 120 mmHg	Target SBP of < 120 mmHg showed ↓ CVD events vs. < 140 mmHg; however, women were underrepresented (36%)
Adults > 80 years (HYVET trial)*	< 150 mmHg	Indapamide (with or without perindopril) reduced stroke, mortality, CHF; mean age of 83 with 61% women enrolled
Chinese Adults 60–80 years (STEP trial)*	110–130 mmHg	Target SBP of 110–130 mmHg showed ↓ CVD events when compared to 130–150 mmHg

*Very frail individuals were excluded from the SPRINT, HYVET, and STEP trials

Managing HTN pharmacologically in older women can be complicated by polypharmacy, drug-drug interactions, age-related cognitive decline, and heightened susceptibility to adverse effects such as orthostatic hypotension. Polypharmacy–defined as concurrent use of five or more medications–is common in older adults, especially women, who often live longer than men and are more likely to have multiple chronic conditions. Moreover, women may experience higher rates of medication-related side effects–such as ACEi-induced cough, diuretic-related hyponatremia or hypokalemia, and peripheral edema from CCBs [143–146]. Nevertheless, certain therapies may offer dual benefits in this population. Thiazide diuretics, for example, may be particularly advantageous in older women with osteopenia or osteoporosis due to their bone-sparing properties and associated reduction in fracture risk. In addition, fixed-dose combination pills can streamline medication regimens, improve adherence and reduce pill burden.

As women continue to live longer and comprise a growing proportion of the aging population, individualized and evidence-informed approaches to BP management are increasingly necessary. Research aimed at defining optimal targets and best practices for older women remains a critical priority to improve long-term CV outcomes and support healthy aging.

## Special Populations and Health Disparities

### Racial and Ethnic Disparities

#### Disproportionate Burden of HTN in Black, Hispanic, and Indigenous Women

Non-Hispanic Black (NHB) women face one of the highest global HTN rates, with a prevalence of 55.3%, and are less likely to achieve BP control compared to non-Hispanic White women [145]. Data on Hispanic/Latina subgroups are more variable, though AHA statistics report a prevalence of 40.8% [147]. Among American Indian/Alaska Native populations, HTN remains a major CVD risk factor, especially among those with DM, with prevalence estimates ranging from 25 to 41% [147]. Nationally, HTN control remains suboptimal, with fewer than 25% of adults achieving target BP [148]. Although race and ethnicity are social constructs, these disparities reflect deeper structural inequities, systemic racism, and unmet social needs.

#### Social Drivers of Health and Access to Care

**Fig. 2:**
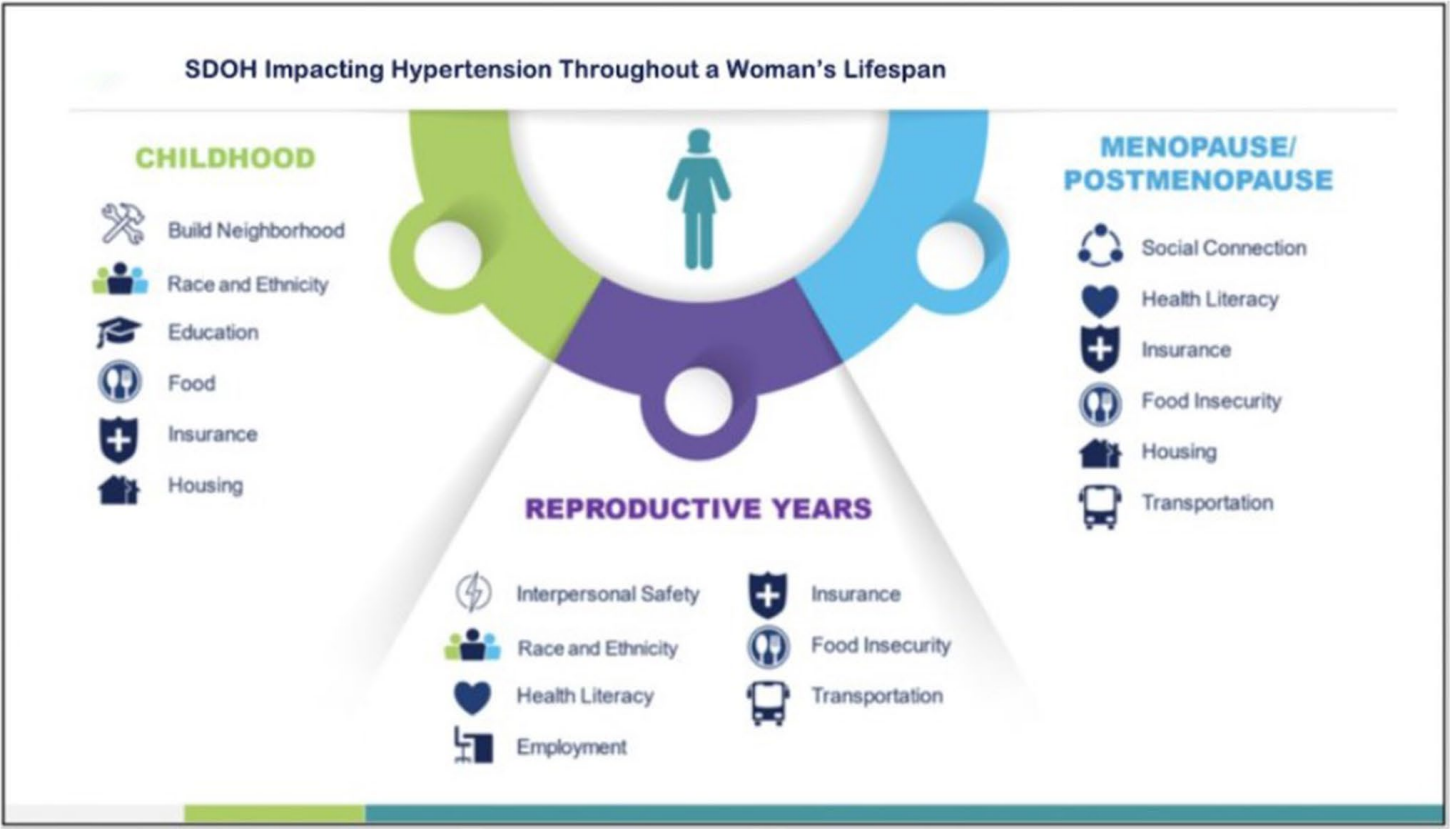
SDOH impacting hypertension throughout a Woman’s Lifespan

Although long described as “social determinants of health”, these factors are now increasingly referred to as “social drivers of health,” a preferred term that highlights their modifiable and actionable nature. Hypertension outcomes are strongly shaped by these drivers–the conditions in which people live, work, and age [149, 150]. They remain central contributors to CV disparities; evidence shows that adjusting for SDOH can reduce or even eliminate racial gaps in CV mortality and outcomes [150]. Recognizing this shift in terminology underscores the importance of addressing these upstream, structural factors to improve HTN control across the lifespan of women (Fig. 2).

Emerging studies now focus on the cumulative impact of multiple adverse SDOH on CVD. Composite indices demonstrate how intersecting factors–such as low income, housing instability, or lack of insurance–worsen outcomes [151, 152]. In the Reasons for Geographic and Racial Differences in Stroke (REGARDS) Study, adults with three or more adverse SDOH had a 1.5-fold increased stroke risk versus those with none [151]. In Black adults, having four or more adverse SDOH increased apparent treatment-resistant HTN (aTRH) risk by 70%; for White adults, the risk more than doubled [151]. Other national datasets, including analyses of disaggregated Asian groups, echo these findings: cumulative SDOH burden worsens cardiometabolic health [152]. Access to healthcare–especially where income, geography, or insurance limit options– remains a key determinant [153] Women with low socioeconomic status or limited healthcare access are less likely to receive preventive services and more likely to delay or avoid care, contributing to poorer outcomes [154].

### Socioeconomic Factors and Rural Populations

#### Barriers to Care and Adherence

Lifestyle interventions are foundational to HTN prevention and treatment, yet evidence-based strategies remain underutilized in racial and ethnic minority groups [155]. Barriers include unmet SDOH needs, clinician bias, systemic racism, and clinical inertia [156]. Under-resourced clinics, policy inequities, and poor care coordination further widen the gap [156, 157]. Clinical inertia–failure to start or adjust treatment–is often tied to bias and uncertainty [157]. For example, minority patients may face delays in medication changes due to concerns about adherence or implicit bias. Rural populations face additional issues, including fewer primary and specialty care options, leading to worse chronic disease outcomes, including HTN [158].

#### Tailored Community-Based Interventions

Despite ongoing gaps in HTN care–especially among NHB and Indigenous women– traditional models often fall short. Community-based strategies can bridge this gap. Interventions tailored to cultural norms and community strengths have improved BP awareness, treatment, and control. These models promote trust, empower patients, and advance equity in HTN care (Table 5).

**Table 5:** Community-based tailored hypertension interventions

Pitfalls	Tailored intervention
Inaccurate BP Measurement	Promote self-measured BP monitoring in patient-centered models to improve accuracy and patient engagement
Lack of Optimization or Embracing Technology	Leverage patient portals and mobile health apps to enhance engagement and self-management
Explicit and Implicit Bias	Adopt culturally humble approaches, implement anti-bias training, and validate patient concerns
Lack of Ongoing Community Engagement	Build partnerships with trusted community organizations (e.g., salons, churches) to foster engagement
Therapeutic Inertia in Hypertension Treatment	Utilize standardized BP management protocols (e.g., SPRINT) to promote equitable treatment intensification
Inadequate or Lack Health Insurance Coverage	Policies to reduce or eliminate patient out-of-pocket costs of antihypertensive medications improves hypertension control
Cost-Related Barriers	Advocate for policies to reduce out-of-pocket costs for antihypertensives
Lack of Team-Based Care	Implement team-based care with physicians, nurses, pharmacists, and community health workers
Failure to Assess and Address Social Drivers of Health	Collaborate with community-based organizations to align goals and address SDOH

## Emerging Trends and Future Directions

### Precision Medicine and Sex-specific Therapies

HTN affects women differently than men across their lifespan. Women experience higher rates of adverse CV outcomes—including acute MI, diastolic dysfunction, arterial stiffness, chronic kidney disease, left ventricular hypertrophy [159], and left atrial enlargement [160] —at lower systolic and diastolic BP levels than men [161]. As previously mentioned, they are also more likely to experience side effects from standard prescribed antihypertensive therapies [1]. Despite this, most current guidelines—including the 2025 ACC/AHA [1] and 2024 European Society of Cardiology/European Society of Hypertension (ESC/ESH) [162] recommendations—do not include sex-specific thresholds for diagnosis or treatment. Notable progress has been made in pregnancy-related HTN care. The 2022 CHAP trial showed that lowering the treatment thresholds to 140/90 mmHg in pregnant individuals with chronic HTN significantly reduces the risk of adverse outcomes without impacting fetal growth [50]. As a result, ACOG revised its guidelines to reflect this threshold [51], and the 2024 ESC guidelines extended the ≥ 140/90 mmHg treatment recommendation to all pregnant individuals, including those with gestational HTN and preeclampsia [160]. These changes underscore the value of advancing sex-specific BP targets beyond pregnancy.

### Remote Monitoring and Digital Health Innovations

Wearable and remote BP monitoring technologies provide new opportunities for personalized and accessible care. Although current guidelines do not endorse wearable devices for home BP monitoring due to concerns about accuracy, they nonetheless support home BP monitoring with validated devices, especially when combined with multidisciplinary care approaches and simplified regimens to improve adherence and outcomes.

Emerging techniques such as wrist cuffs, ECG-based sensors, and photoplethysmography (PPG) wearables allow for home-based BP tracking [163–167]. These tools may be particularly beneficial for women, who often have limited time or access to in-person visits because of caregiving or work responsibilities. Remote monitoring is also valuable in the perimenopausal and later life periods, enabling early risk identification and long-term management.

Despite this promise, most clinical guidelines continue to recommend validated upper-arm cuffs for both office and home use, citing ongoing concerns about the reliability of newer devices [1, 160, 168]. The most recent ACC/AHA and ESC/ESH guidelines endorse wrist cuffs only when arm-based measurement is not feasible, and they do not recommend cuffless options to monitor HTN in any clinical scenario [1, 160, 169]. Establishing standardized validation protocols for wearable and cuffless technologies, and their integration into clinical guidelines, remains an important unmet need.

### Research Gaps and Policy Implications

Significant gaps remain in understanding sex-based differences in HTN pathophysiology, drug dosing, and treatment response across the lifespan [160]. Women–particularly those in reproductive, menopausal, and postmenopausal stages–remain underrepresented in research. As noted earlier, the SPRINT trial highlights this ongoing challenge, with limited enrollment and focus on women in cardiovascular studies [140]. This underrepresentation constrains our understanding of how HTN develops and progresses in female populations. For HDP, clear diagnostic thresholds for out-of-office BP monitoring are still lacking, and management continues to rely heavily on clinic-based measurements [169]. Emerging technologies, such as intermittent or continuous cuffless BP monitors, have not yet been adequately evaluated in diverse, real-world female populations [170]. Future research must include women across age, reproductive stage, race, ethnicity, and socioeconomic status to close persistent knowledge and treatment gaps [171].

### Future Directions

Team-based care remains a proven, scalable strategy for improving HTN outcomes across a woman’s lifespan. Coordinated HTN management programs–embedded within primary care, cardiology, nephrology, or cardio-obstetrics–enhance outcomes when delivered through multidisciplinary teams [170]. Nurses, midwives, pharmacists, emergency medical technicians (EMTs), doulas, community health workers, and behavioral health specialists all contribute to BP screening, education, medication adherence, and lifestyle counseling. Their involvement is especially vital in resourced-limited communities where access and continuity of care are often barriers.

Innovative models–including community-based programs, virtual care models, and tailored pathways for postpartum or menopausal care–can improve engagement and outcomes [170, 172, 173]. We are learning that such approaches are particularly critical in rural settings, where maternal morbidity remains higher than urban areas [173]. Recent studies, such as ESPRIT (Effects of Intensive Systolic Blood Pressure Lowering Treatment in Reducing Risk of Vascular Events), BPROAD (Blood Pressure Control Target in Diabetes), and CRHCP (China Rural Hypertension Control Project) have shown that intensive BP control, along with community support programs, reduce vascular events [174–176]. However, these studies were mainly conducted in China and would benefit from further research to improve generalizability.

As HTN care continues to evolve, closing persistent gaps in CV health will require the integration of sex-specific science, digital tools, and interdisciplinary collaboration. While more research is needed, recent initiatives–such as the ACC’s “*Monitoring Blood Pressure Throughout a Woman’s Lifecycle,*” a comprehensive collection of learning tools–represent an important step toward expanding awareness, equity, and precision in women’s CV care [177].

## Conclusion

HTN in women is a dynamic, lifelong condition influenced by hormonal transitions, reproductive factors, psychosocial stressors, and structural inequities. BP regulation shifts significantly across life stages–from adolescence, pregnancy, and menopause to older age–each introducing unique risks and treatment considerations.

Despite growing awareness, major research and clinical gaps remain–especially regarding lifestyle stressors, caregiving burdens, and the lived experiences of women of color. While progress has been made in pregnancy and geriatric care, current guidelines still lack comprehensive sex-specific approaches to diagnosis and treatment.

A personalized, life-course strategy that integrates team-based care, community engagement, digital health tools, and equitable research is essential to improve HTN outcomes for all women.
